# Vertical transmission does not always lead to benign pathogen–host associations

**DOI:** 10.1093/evlett/qrad028

**Published:** 2023-08-10

**Authors:** George Shillcock, Francisco Úbeda, Geoff Wild

**Affiliations:** Department of Mathematics, Western University, London, Canada; Department of Biological Sciences, Royal Holloway, University of London, Egham, Surrey, United Kingdom; Department of Mathematics, Western University, London, Canada

**Keywords:** commensalism, evolutionary epidemiology, infectious disease, microparasite

## Abstract

Understanding the capacity of pathogens to cause severe disease is of fundamental importance to human health and the preservation of biodiversity. Many of those pathogens are not only transmitted horizontally between unrelated hosts but also vertically between parents and their progeny. It is widely accepted that vertical transmission leads to the evolution of less virulent pathogens, but this idea stems from research that neglects the evolutionary response of hosts. Here, we use a game-theory model of coevolution between pathogen and host to show that vertical transmission does not always lead to more benign pathogens. We highlight scenarios in which vertical transmission results in pathogens exhibiting more virulence. However, we also predict that more benign outcomes are still possible (a) when generating new horizontal infections inflicts too much damage on hosts, (b) when clearing an infection is too costly for the host, and (c) when vertical transmission is promoted by a greater growth rate of the host population. Though our work offers a new perspective on the role of vertical transmission in pathogen–host systems, it does agree with previous experimental work.

## Introduction

Some pathogens exploit their host by prudently taking up resources without causing disease, while others exploit their host imprudently, causing diseases that may result in the death of their host (Frank, 1996). Virulence refers to the relative capacity of a microbial pathogen to cause disease of a given severity (Casadevall & Pirofski, 2001; Frank, 1996). Efforts to understand why some pathogens evolve to be more virulent than others are of importance to human health, agricultural practice, and the structure and function of ecosystems (Geoghegan & Holmes, 2018; Lafferty et al., 2008; Mennerat et al., 2010). These efforts have shown that virulence is sensitive to life-history details of both pathogen and host, on evolutionary timescales. These details include, but are not limited to, the social behavior of hosts, the immune response of hosts, the competition between pathogen strains within hosts, and the novelty of the host for the pathogen (Ewald, 1994; Frank, 1992; Stearns & Medzhitov, 2015).

Yet another factor known to affect virulence is the way a pathogen is transmitted from one host to another. Generally speaking, pathogen transmission can be partitioned into two modes: vertical and horizontal. Vertical transmission occurs when pathogens are transferred directly from a parent host to their progeny (Ebert, 2016; Fine, 1975). Vertical transmission occurs, for example, when pathogens are transmitted across the placenta, through breastfeeding, and through the germ line, in the course of offspring feeding on eggshells or (for single-celledorganisms) via cytoplasmic inheritance (Ewald, 1994; Fine, 1975; Mims, 1981). Horizontal transmission occurs in all other cases—that is, when pathogens are transferred between hosts except when the transfer occurs directly from parent to progeny (Ebert, 2016; Fine, 1975).

It is widely accepted that vertical transmission leads to the evolution of less virulent pathogens, as transmission from parent to progeny rewards pathogens that inflict less harm on their hosts (Cressler et al., 2016; Dieckmann et al., 2002; Ebert, 2016; Ferdy & Godelle, 2005; Frank, 2002, 1996; Lipsitch et al., 1996; Schmid-Hempel, 2021; Stearns, 2012; Stearns & Medzhitov, 2015; Úbeda & Jansen, 2016). A number of empirical studies also show that enhancing vertical transmission can reduce pathogen virulence (Bull et al., 1991; Bull and Molineux, 1992; Stewart et al., 2005; Tintjer et al., 2008).

Despite existing theoretical and experimental work, we cannot link the evolution of benign pathogen–host associations inextricably to vertical transmission for at least two reasons. First, there exist examples of highly virulent, vertically transmitted pathogens (Fries & Camazine, 2001; Kover et al., 1997; Lipsitch et al., 1995). While it is possible that such pathogens would be even more virulent in the absence of vertical transmission, the absolute severity of infections suggests that vertical transmission does not always temper the effects of a disease, in a practical sense. Second, experimentally induced vertical transmission has been implicated in the evolution of host countermeasures. For example, Pagán et al. (2014) found controlled vertical transmission of a plant virus led to the evolution of host resistance, observed as lower within-host pathogen replication rates. If lower rates of within-host replication extend the infectious period, we might predict the subsequent evolution of increased virulence in response to enhanced opportunities for horizontal transmission, outside of a controlled laboratory setting (Fleming-Davies et al., 2018; Gandon et al., 2001; Read et al., 2015). Thus, host countermeasures could, in principle, direct coevolutionary trajectories away from benign outcomes if they limit a pathogen’s ability to transmit vertically or if they reduce the overall duration of parasitic infections themselves. The evolution of host responses to vertical disease transmission, in particular, has been neglected by modeling efforts to date.

Our goal is to explore the extent to which a pathogen’s ability to transmit vertically results in more benign pathogens, from a theoretical perspective. We adopt a coevolutionary modeling framework and so distinguish ourselves from previous theoretical work on vertical transmission and virulence (Ferdy & Godelle, 2005; Lipsitch et al., 1996; Úbeda & Jansen, 2016). Our modeling predicts that vertical transmission does not always reduce the virulence that stems from parasitic infections. We find, specifically, benign outcomes tend to correspond to scenarios in which horizontal transmission among hosts is less profitable, host immune function is more expensive, and the intrinsic rate of host population growth is high. Our findings suggest that broad claims about the tempering effect of vertical transmission should, themselves, be tempered and re-evaluated with greater consideration given to host biology.

## Methods

### Background

Our model extends one originally proposed by Day and Burns (2003). A detailed explanation is provided in the accompanying Supplementary Material.

We consider a population comprised of individuals that play host to an infectious pathogen. Hosts not infected by the pathogen are susceptible to infection; they give birth at per-capita rate $ b_S $ and die at per-capita rate $\mu$. In the absence of the pathogen, then, the net (intrinsic) rate of host population growth is $ b_S -\mu>0$.

For their part, hosts infected by the pathogen die at per-capita rate $\mu+\alpha$, where $\alpha$ represents the rate of pathogen-induced mortality. These same hosts recover from infections at per-capita rate $\gamma$ and, when they do, we assume that they are immediately susceptible to reinfection. We also assume that recovery diverts resources away from reproduction, so infected hosts reproduce at per-capita rate $b_{i}(\gamma )=b_{S}e^{-\lambda \;\gamma^{2}/b_{S}}$, where the constant $\lambda$ reflects the cost of host immune function. For $\lambda\ll b_S $, we get $b_{i}(\gamma )\approx b_{S}-\lambda \gamma^{2}$, and we use this approximation going forward.

The pathogen in our model creates new infections both horizontally and vertically. We assume new horizontal infections are created at an overall rate that is proportional to the product of infected and uninfected host numbers. The constant of proportionality here is modeled as $\beta (\alpha )=m\alpha^{n}$, for constants $ m >0$ and $ 0< n <1$. As our notation suggests, we treat greater pathogen-induced host mortality as a necessary consequence of an increased rate of horizontal transmission. The constant *n* represents the percent increase in $\beta(\alpha)$ owing toa 1% increase in $\alpha$.

Following previous authors (Lipsitch et al., 1996; Úbeda & Jansen, 2016), we assume that new infections arise vertically with probability *v* whenever an infected host gives birth. Therefore, infected hosts produce new infected hosts at per-capita rate $vb_{i}(\gamma )$ and produce new uninfected hosts at per-capita rate$\left(1-v)b_{i}(\gamma \right)$.

If *S* is the number of susceptible hosts in the population at a given time, and is the number of infected hosts in the population at the same time, then the description above can be restated mathematically as


$$\begin{array}{l} S\prime =b_{S}S+(1-v)b_{i}(\gamma )I-\beta (\alpha )SI-\mu S+\gamma I,\\ I\prime =vb_{i}(\gamma )I+\beta (\alpha )SI-(\mu +\gamma +\alpha )I \end{array}$$ (1)

where the prime indicates differentiation with respect to time. It is not obvious from Equation 1 that we assume a pathogen can create new vertical infections without harming its host. Though this particular assumption is consistent with previous theoretical work (Lipsitch et al., 1996; Úbeda & Jansen, 2016), it is likely an oversimplification. Specifically, the assumption means that we expect our model to overemphasize any mitigating effects vertical transmission may have on pathogen–host antagonism.

### Pathogen–host coevolution

We consider pathogen-induced mortality $\alpha$ to be a trait expressed by the pathogen, and recovery rate $\gamma$ to be a trait expressed by the host. We model the coevolution of these two traits as the solution to a simultaneous-move game played between a pathogen and its host. The pathogen’s goal in the game is to find an $\alpha$ value that maximizes its fitness given the $\gamma$ expressed by its host. Similarly, the host’s goal in the game is to find a $\gamma$ value that maximizes its fitness given that $\alpha$ expressed by its pathogen. In Supplementary Material, we show that the pathogen’s best response to its host, called $\alpha_{\text{br}}(\gamma)$, is the $\alpha$ that satisfies


$$\beta \prime (\alpha )=\frac{\beta (\alpha )}{\mu +\alpha +\gamma -vb_{i}(\gamma )}.$$ (2a)

We also show that the host’s best response to its pathogen, called $\gamma_{\text{br}}(\alpha)$, is the $\gamma$ that satisfies


$$b_{i}\prime (\gamma )=\frac{\left(\gamma )-(\mu +\alpha \right)}{(\mu +\alpha )(1-v)+\gamma }.$$ (2b)

**Figure 1:**
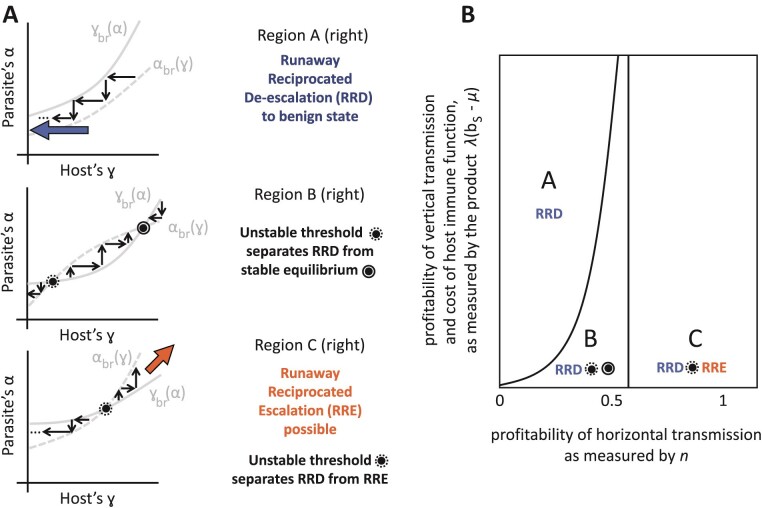
Coevolutionary outcomes predicted by our model when vertical transmission is certain, $ v =1$. (A) Solutions to the system of equations in 2a and 2b can be found wherever best-response curves $\alpha_{\text{br}}(\gamma)$ and $\gamma_{\text{br}}(\alpha)$ intersect. Solutions may correspond to a stable equilibrium (dot surrounded by solid circle) or an unstable threshold (dot surrounded by dashed circle). Thresholds separate benign runaway reciprocated de-escalation from less benign outcomes like runaway reciprocated escalation. (B) We divide parameter space into three regions separated by the curve $n^{2}+8\lambda (b_{S}-\mu )(n-1/2)=0$ and the vertical line through $ n =1/2$. In region A only runaway reciprocated de-escalation is possible. In region B, we find a threshold solution separates runaway reciprocated de-escalation from a relatively escalated stable equilibrium outcome. In region C, a threshold separates mutual de-escalation from runaway reciprocated escalation.

Solving Equations 2a and 2b gives a pair of values $(\alpha^{\ast},\gamma^{\ast})$ that represents a candidate coevolutionary outcome. The candidate pair is a stable outcome (Nash equilibrium or simply a “stable equilibrium”) whenever $\alpha_{\text{br}}\prime(\gamma^{\ast})\, \gamma_{\text{br}}\prime(\alpha^{\ast})<1$, meaning that the neither party can improve its fitness by unilaterally deviating from $(\alpha^{\ast},\gamma^{\ast})$ (Figure 1). In Supplementary Material, we compare this condition to familiar evolutionary stability concepts. We show that traits comprising a stable equilibrium, as defined here, are Evolutionarily Stable in that they cannot be invaded by alternatives (Maynard Smith, 1982). We also show that the stable equilibrium is convergence stable in both the regular sense (e.g., Abrams et al., 1993) and the absolute sense (Kisdi, 2006). Absolute convergence stability, in particular, means that discrepancies in the mutation rates of coevolving parties do not interfere with our conclusions (Kisdi, 2006).

When $\alpha_{\text{br}}^\prime(\gamma^{\ast})\, \gamma_{\text{br}}^\prime(\alpha^{\ast})>1$, the pair $(\alpha^{\ast},\gamma^{\ast})$ is as an unstable equilibrium and acts as a threshold that separates two distinct outcomes from one another. On one hand, when the rate of pathogen-induced mortality is low and the host’s immune response is mild, our model predicts coevolution toward a benign, mutually de-escalated end state (e.g., Figure 1A; see also Table 1). We refer to this outcome as “runaway reciprocated de-escalation.” On the other hand, if either the pathogen induces a high rate of mortality or the host mounts an overly aggressive immune response, our model predicts mutual escalation of aggression, possibly to some stable equilibrium level. When no equilibrium exists (stable or unstable), escalation could proceed indefinitely, a situation we call “runaway reciprocated escalation”(e.g., Figure 1A; see Table 1).

**Table 1:** Glossary of terminology related to coevolutionary outcomes for $\alpha$ and $\gamma$.

Terminology	Explanation
Runaway reciprocated escalation	Selection leads pathogen and host to increase their respective trait values indefinitely. The extent of escalation, here, is limited only by model assumptions. We consider this to be the most aggressive coevolutionary outcome. We also consider pathogen–host coevolution to be more aggressive when this outcome is more easily accessed by selection (e.g., as threshold, defined below, falls).
Runaway reciprocated de-escalation	Selection leads pathogen and host to reduce their respective trait values indefinitely. De-escalation is limited either by the boundary of phenotype space or by model assumptions. We consider this to be the most benign coevolutionary outcome. We also consider pathogen–host coevolution to be more benign when this outcome is more easily accessed by selection (e.g., as threshold, defined below, increases).
Threshold	An intersection between best-response curves, but not a coevolutionary outcome. A threshold separates runaway reciprocated de-escalation from either a stable equilibrium (defined below) outcome or reciprocated runaway escalation. In other words, it establishes the basin of attraction for relatively benign and relatively aggressive coevolutionary outcomes. We associate rising thresholds with more benign outcomes as the rise increases the scope for runaway reciprocated de-escalation. We associate falling thresholds with more aggressive outcomes as, in this case, the scope for more aggressive, stable equilibrium outcomes or runaway reciprocated escalation is increased.
Stable equilibrium	An intersection between best-response curves that satisfies the stability condition presented in the text. We consider this outcome to be more aggressive than runaway reciprocated de-escalation but not as aggressive as runaway reciprocated escalation. We recognize that exactly how aggressive this outcome is can change as model parameters change. We have introduced some extra terminology to describe these changes: reciprocated de-escalation, unreciprocated de-escalation, and reciprocated escalation (see below).
Reciprocated de-escalation	Used to describe a shift in the stable equilibrium outcome of coevolution owing to a change in model parameters (often ). Here, the equilibrium shifts because both the pathogen and the host trait values have become lower relative to before the parameter in question was changed. We consider the shift in the equilibrium outcome to indicate a more benign outcome, again relative to before the parameter in question was changed.
Unreciprocated de-escalation by pathogen	Used to describe a shift in the stable equilibrium outcome of coevolution owing to a change in model parameters (often ). Here, the equilibrium shifts because the pathogen lowers its trait value, but the host increases its trait value relative to before the parameter in question was changed.
Reciprocated escalation	Used to describe a shift in the stable equilibrium outcome of coevolution owing to a change in model parameters (often ). Here, the equilibrium shifts because both the pathogen and the host trait values have become greater relative to before the parameter in question was changed. We consider the shift in the equilibrium outcome to indicate a more aggressive outcome, again relative to before the parameter in question was changed.

### Virulence

We work with three definitions of pathogen virulence. Our first definition is simply the rate of pathogen-induced mortality, $\alpha$. Previous authors have adopted this definition because it is convenient (Day and Burns, 2003; Úbeda & Jansen, 2016). The quantity $\alpha$, alone, does not capture the possibility that disease severity is also related to the interplay between pathogen traits and those of the host (Casadevall & Pirofski, 2001; Day, 2002; Read, 1994). To address this shortcoming, our second definition combines $\alpha$ with host recovery rate $\gamma$ into one expression for case mortality,


$$\frac{\alpha }{\alpha +\gamma +\mu }.$$ (3)

Case mortality can be interpreted as the probability with which an infected host dies for reasons owing to its infection. It has proven to be a useful measure of virulence in other models of pathogen–host evolution (Day, 2002). Our third definition also combines pathogen and host traits, but with this definition, we cast virulence as the reduction in host fitness owing to infection (Lipsitch et al., 1995, 1996). In Supplementary Material, we show that


$$\frac{\mu +\alpha -b_{i}(\gamma )}{\mu +\alpha +\gamma -vb_{i}(\gamma )}$$ (4)

expresses the difference between the fitness of a susceptible host and an infected one in our model.

## Results

### Special extreme cases

If vertical transmission is absent ($ v =0$), our model reduces to that studied by Day and Burns (2003). In this case, we can solve Equations 3 and 4 to find $\alpha^{\ast}=\alpha_{0}^{\ast}$ and $\gamma^{\ast}=\gamma^{\ast}_{0}$ where,


$$\alpha_{0}^{*}=\frac{(2\lambda \mu +1)n+\sqrt{{\left(2\lambda \mu +1\right)}^{2}-(1{-}^{2})(4\lambda b_{S}+1)}}{2\lambda (1{-}^{2})/n},$$ (5a)

$$\gamma_{0}^{*}=\frac{1-n}{n}\alpha_{0}^{*}-\mu .$$ (5b)

The results, here, coincide with the predictions in Day and Burns (2003), though our notions of evolutionary stability differ (see Supplementary C.2).

High rates of vertical transmission are not uncommon in some pathogens, with the proportion of offspring infected reaching 100% in some reported cases (Tintjer et al., 2008; Wallace & Drake, 1962). When $ v =1$, we can again solve Equations 2a and 2b, but now we find two candidate equilibrium outcomes. These candidates are characterized by $\gamma^{\ast}=\gamma^{\ast}_{1}$ and $\gamma^{\ast}=\gamma^{\ast}_{2}$, where


$$\gamma_{1}^{*}=\frac{-n+\sqrt{8\lambda (b_{S}-\mu )(n-1/2)+n^{2}}}{4(n-1/2)\lambda },$$ (6a)

$$\gamma_{2}^{*}=\frac{-n-\sqrt{8\lambda (b_{S}-\mu )(n-1/2)+n^{2}}}{4(n-1/2)\lambda }.$$ (6b)

Of course, $\alpha_{1}^{\ast}=\alpha_{\text{br}}(\gamma_{1 }^{\ast})$ and $\alpha_{2}^{\ast}=\alpha_{\text{br}}(\gamma_{2 }^{\ast})$ give the corresponding pathogen trait, expressed in terms of the pathogen’s best response to the appropriate equilibrium host trait. In this case, the pathogen’s best response is expressed as


$$\alpha_{br}(\gamma )=\frac{n}{1-n}(\lambda \gamma^{2}+\gamma -(b_{S}-\mu )).$$ (6c)

Of the two candidate pairs, only $(\alpha_{2}^{\ast},\gamma_{2}^{\ast})$ is a stable equilibrium. At this equilibrium, we can ensure that $b_{i}(\gamma )$ is positive by choosing $ b_S >\lambda\,\gamma^{2}$.

Extensive numerical comparisons suggest that the stable equilibrium with $ v =1$, when it exists, is escalated compared with the $ v =0$ case all else being equal, meaning that $\alpha_{0}^{\ast}<\alpha_{2}^{\ast}$ and $\gamma_{0}^{\ast}<\gamma_{2}^{\ast}$ (see Supplementary Table C.1). Based on the comparison at this (admittedly broad) scale, we can say that vertical transmission does not inevitably lead to lower levels of pathogen-induced mortality, nor does it inevitably lead to lower rates of host recovery. A similar broad-scale comparison suggests complete vertical transmission results in marked virulence when this quantity is measured as reduction in host fitness as in Equation 4 (see Supplementary Table C.2).

We would like to know how the parameters of our model affect coevolutionary outcomes when infections can be transmitted vertically. To help our understanding in this regard, we identify three regions of parameter space, each supporting a distinct set of outcomes for the case $ v =1$ (Figure 1B). In the first region, marked A in Figure 1B, no pair of real numbers satisfies Equations 2a and 2b simultaneously. Thus, both $\alpha$ and $\gamma$ tend to low levels as coevolution proceeds in this case (“runaway reciprocated de-escalation”). As we pass from A in Figure 1B into B, both the threshold pair $(\alpha_{1}^{\ast},\gamma_{1}^{\ast})$ and the stable equilibrium $(\alpha_{2}^{\ast},\gamma_{2}^{\ast})$ appear. Moving from A to B then raises the possibility that coevolution leads to more aggressive pathogens and hosts. The potential for even more aggressive outcomes is raised as we move from B to C, as in region C only the threshold exists and it separates relatively benign outcomes from “runaway reciprocated escalation.”

What lessons can we take away from Figure 1? Because region A increases in size at the expense of region B, we predict that benign coevolutionary outcomes are promoted as (a) the profitability of horizontal transmission *n* decreases, (b) the cost of host recovery $\lambda$ increases, and (c) the intrinsic rate of increase in the host population $ b_S -\mu$ increases. Point (a) is immediately understandable: If horizontal transmission becomes less effective, then a pathogen that exploits its host more readily to create more, new horizontal infections will be at a disadvantage. Reduction in exploitation would then be reciprocated by the host, leading to de-escalation. Point (b) is also easily understood: If it costs a host more to rid itself of its pathogen then it will not be able to afford a quick recovery. Slower host recovery would then be reciprocated by the pathogen, again leading to de-escalation. Point (c) can be understood if we recognize that when $ b_S -\mu$ is high, the vertical spread of the pathogen is enhanced. In other words, vertical transmission becomes more profitable. Since we assume that host exploitation is not required for vertical transmission to be successful, we of course observe de-escalation to benign outcomes as the vertical mode becomes a more attractive option.

Similar predictions and similar lessons emerge when we focus attention on the candidate outcomes in region B of Figure 1. As we explain in Section C of Supplementary Material, we connect more benign outcomes in Region B to parameter combinations that increase the threshold $(\alpha_{1}^{\ast},\gamma_{1}^{\ast})$. We also connect more benign outcomes to parameter combinations that de-escalate the stable equilibrium $(\alpha_{2}^{\ast},\gamma_{2}^{\ast})$. That said, benign outcomes in B specifically are once again promoted by lower *n*, larger $ b_S -\mu$, and larger $\lambda$ (see Supplementary Figure C.2).

In Region C, only the threshold $(\alpha_{1}^{\ast},\gamma_{1}^{\ast})$—the one that separates runaway reciprocated de-escalation from runaway reciprocated escalation—exists. In this region, then, we associate more benign outcomes with increases in the threshold, as described above. We find that benign outcomes here are promoted when *n* is lowered and $ b_S -\mu$ raised (Supplementary Figure C.3). Notably, the cost parameter $\lambda$ interacts with and $ b_S -\mu$ to affect coevolutionary outcomes in region C. Whereas increases in cost in Regions A and B uniformly promote more benign outcomes, the opposite is true in Region C when *n* is large and/or $ b_S -\mu$ is small (Supplementary Figure C.3).

### Generic intermediate cases

**Figure 2:**
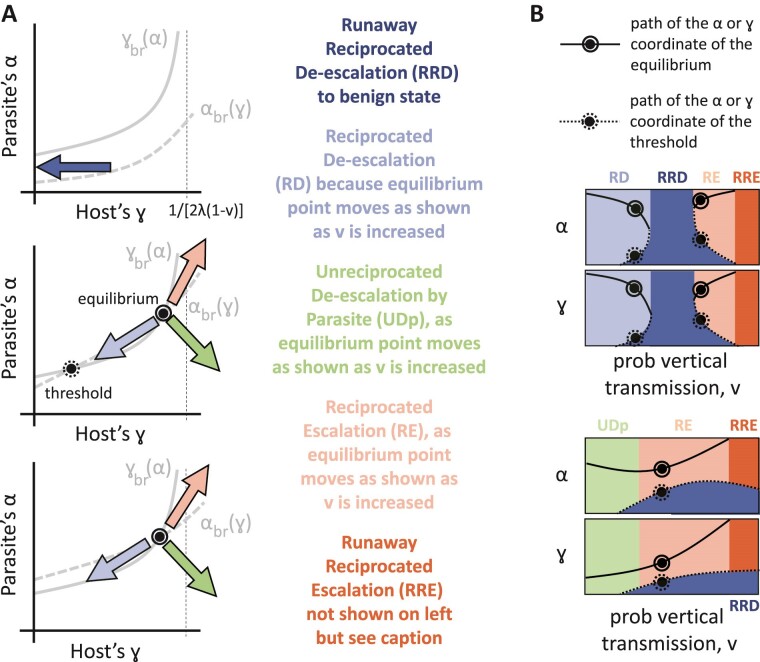
Coevolution outcomes predicted when the probability of vertical transmission is intermediate, $ 0< v <1$. We can now classify outcomes in greater detail by considering how the stable equilibrium (and threshold too) responds to changes in model parameters. We can envision the new categories of outcome as either (A) being defined by shifts in the position of the stable equilibrium in $\gamma,\alpha$-space induced by increases in a parameter or (B) with specific attention paid to the parameter *v*, the shape of the path traced out by the curve of stable equilibrium points, thought of as a function of *v*. One pattern that is noticeably absent from either panel is one where pathogen’s stable equilibrium $\alpha$ increases with but host’s stable equilibrium $\gamma$ decreases with *v*. We stress that, technically, runaway reciprocated escalation is not predicted by our model when $ 0< v <1$. However, in practice, we find that equilibrium $\gamma$ values can sometimes become so large that a key mathematical modeling constraint is violated (see Section C.3 of the Supplementart Material), and we interpret this violation as runaway reciprocated escalation.

In this section, we investigate cases in which the probability of vertical transmission takes values between the two extremes explored above, $ 0< v <1$. In these intermediate cases, we simultaneously solve Equations 2a and 2b numerically (see Supplementary Material). We summarize numerical results by classifying distinct outcomes in a way that connects to the relationship between numerical solutions themselves and model parameters (Figure 2). Though our classification of outcomes is more detailed in this section (see Table 1), the results we report here qualitatively mirror those we reported for $ v =1$.

**Figure 3:**
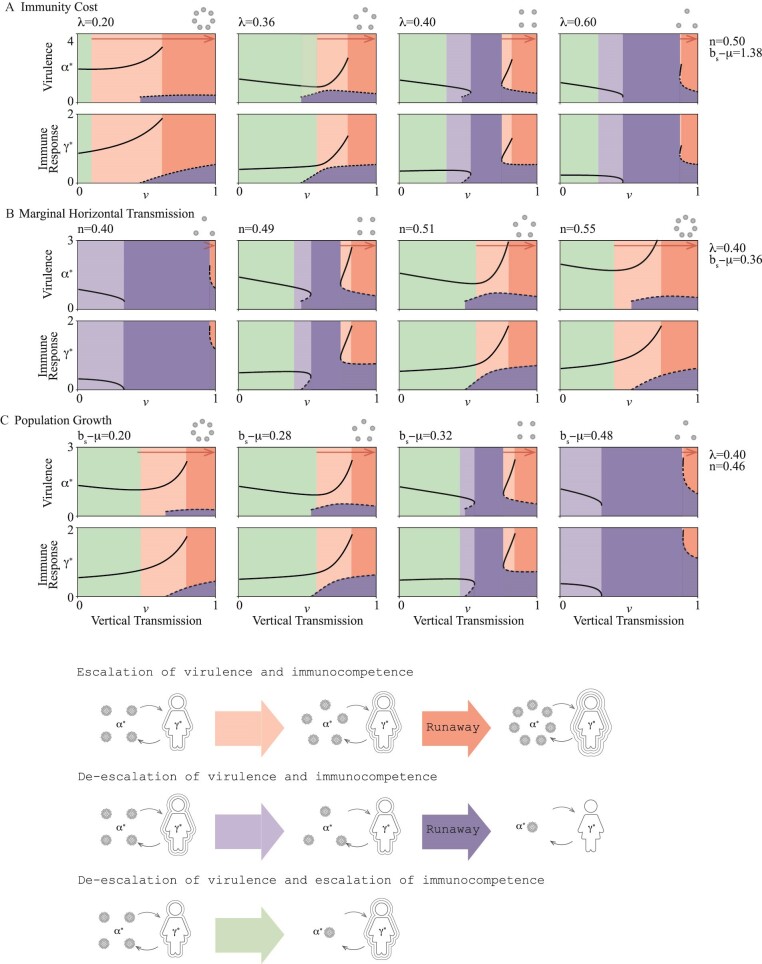
Stable equilibrium trait values (solid lines) and threshold trait values (dashed lines) for pathogen (top row) and host (bottom row), respectively, as the probability of vertical transmission varies. Colors are explained at the bottom of the figure. They correspond to descriptions given in Figure 2 and describe whether trait changes in the pathogen are reciprocated by the host. Parameters for each set of panels (A, B) are shown. Arrows in (A)–(C) highlight the range of values on which $\alpha_{2}{}^{\ast}$ is increasing.

All else being equal, we find that increases to the cost of host immunity, $\lambda$, reduce the level of aggression associated with coevolutionary outcomes. Figure 3i, for example, shows that stable equilibrium trait values tend to fall as $\lambda$ increases, while the scope for runaway de-escalation (captured by thresholds) rises. Specifically regarding the relationship between coevolutionary outcomes and changes to the probability of vertical transmission, we observe a number of things in Figure 3i as $\lambda$ increases:

- runaway reciprocated escalation gives way to “reciprocated escalation,” a situation characterized by stable equilibrium trait values that would both increase in response to a rise in the rate of vertical transmission;
- reciprocated escalation, in turn, gives way to “unreciprocated de-escalation by the pathogen,” characterized by stable equilibrium trait values that would respond differently to a rise in the rate of vertical transmission (specifically, the pathogen’s $\alpha^{\ast}$ would decrease, while the host’s $\gamma^{\ast}$ would increase, if *v* were to rise);
- as reciprocated escalation gives way to “unreciprocated de-escalation by the pathogen, we also see a rise in the often-present possibility of runaway reciprocated de-escalation;
- ‘reciprocated de-escalation,” a situation characterized by stable equilibrium trait values that would both decrease in response to a rise in the rate of vertical transmission, becomes more prominent, as does runaway reciprocated de-escalation.

To put it simply, we see that host immune cost $\lambda$ alleviates the conflict between pathogen and host because “cooler” colors gain prominence moving left to right in Figure 3i. Again, as the cost of immune function rises, an infected host can less afford to rid itself of the pathogen, leaving de-escalation as the only path to mitigating the effects of infection.

We also find that, as *n* increases, horizontal transmission becomes more profitable, and outcomes are more often associated with stronger host defenses and more aggressive exploitation by pathogens. Figure 3ii shows curves of equilibria, thought of as functions of *n*, rising; it also shows curves of thresholds, again thought of as functions of *n*, falling. Furthermore, the figure shows de-escalation giving way to escalation as *n* grows (now, “hotter” colors gain prominence as we move left to right). Relatively aggressive coevolutionary outcomes, here, continue to be driven by the fact that *n* reflects the extent to which pathogens are incentivized to attack their hosts and by extension the extent to which pathogens provoke a more vigorous host response.

Finally, we see that as the intrinsic rate of increase for the host population $ b_S -\mu$ grows, coevolutionary outcomes are more often associated with reduced host defenses and less aggressive exploitation by pathogens (Figure 3iii). As was the case with $\lambda$, we see that greater $ b_S -\mu$ leads to lower curves of equilibria and higher thresholds. We also see escalated outcomes give way to relatively de-escalated ones, again reflecting the fact that increases to $ b_S -\mu$ make vertical transmission a more profitable option than it would be otherwise.

### Other measures of virulence

**Figure 4:**
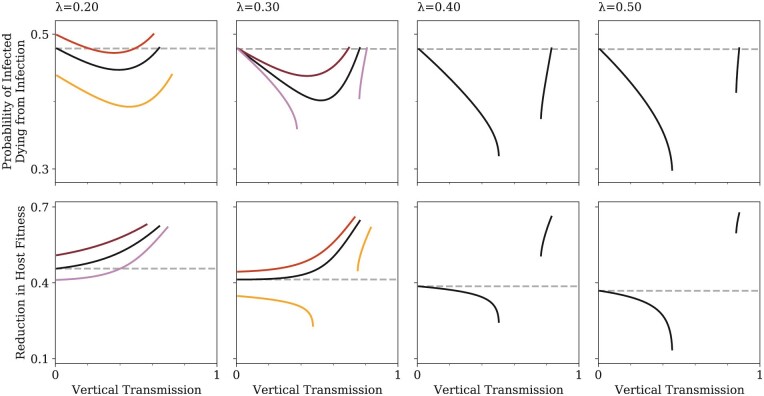
Relationship between case mortality (Equation 3) and the probability of vertical transmission *v* (top panels) and between the reduction in host fitness (Equation 4) and probability of vertical transmission *v* (bottom panels). Immune cost $\lambda$ varies as shown. For black curves $ b_S =1.375$, $\mu=1$, $ n =0.48$. Light orange curves show $ n =0.44$, and dark orange curves show $ n =0.5$. Light purple curves show $ b_S =1.48$, and dark purple curves show $ b_S =1.2$. Dashed horizontal line shows $ v =0$ prediction corresponding to black curves.

So far, we have used the rate of pathogen-induced mortality $\alpha$ to characterize pathogen virulence. We find, however, that coevolutionary outcomes for other virulence measures follow those revealed by studying $\alpha$ alone. Specifically, we again find that virulence, measured as either case mortality or reduction in fitness at stable equilibrium, is (a) reduced as the cost of recovery, $\lambda$, rises, (b) reduced as the intrinsic growth rate of the host, $ b_S -\mu$, rises, and (c) increased as the profitability of horizontal transmission, *n*, rises (Figure 4).

We also examine the consequences of vertical transmission on a broad scale by comparing virulence levels when $ v >0$ to those when $ v =0$. When $ v >0$, we find virulence, defined as case mortality at a stable equilibrium, almost never rises above the level at which it is observed in the absence of vertical transmission, $ v =0$ (Figure 4). By contrast, when $ v >0$ we find virulence, defined as the decrease in host fitness owing to an infection, can rise above the level observed when $ v =0$. The latter finding supports the claim that vertical transmission does not necessarily produce more benign associations between pathogen and host. Indeed, the latter virulence measure is the only one we study that captures impacts on host survival and host reproduction. This feature is important because both survival and reproduction suffer as a result of infection when $ v >0$.

## Discussion

We have used a coevolutionary model to show that vertical transmission does not inevitably lead pathogens and hosts toward more benign outcomes. In agreement with previous work, we find that, all else being equal, vertical transmission does indeed lead to reduced virulence. However, all else is not equal when we allow hosts to also evolve in response to the possibility of vertical transmission. In particular, we find that vertical transmission also gives an infected host extra reason to rid itself of a pathogen. Specifically, vertical transmission extends the fitness consequences of infection for the host beyond survival to include fecundity, as infected offspring are of relatively low quality. Our results show that selection on the host to protect progeny can be enough to drive pathogens in more severe directions notanticipated by previous theory (Cressler et al., 2016; Dieckmann et al., 2002; Ebert, 2016; Frank, 1996, 2002; Ferdy & Godelle, 2005; Lipsitch et al., 1996; Schmid-Hempel, 2021; Stearns, 2012; Stearns & Medzhitov, 2015; Úbeda & Jansen, 2016). Neglecting the possibility of host evolution, as previous theory has done, is not unreasonable. It seems especially appropriate when host–pathogen relationships are relatively new, or host evolution is substantially outpaced by evolution of the pathogen. It follows that the coevolutionary perspective we offer here is best reserved for long-standing host–pathogen relationships or for situations in which host and pathogen traits evolve at comparable rates.

Even though we reject a widely held idea, our work is not at odds with the experimental evidence used to support that idea. In keeping with empirical evidence by Pagán et al. (2014), our model shows that hosts may, in some circumstances, increase their defences despite reduced mortality imposed by vertically transmitted pathogens. Our model also predicts a reduction in pathogen virulence when the return from virulence in terms of horizontal transmission is poor. This matches results obtained by Bull et al. (1991), Bull and Molineux (1992), and Stewart et al. (2005), both of whom found that benevolent interactions between a phage pathogen and its bacterial host were reinforced only when horizontal transmission was curtailed.

We predict benign coevolutionary outcomes are more likely to be associated with vertical transmission when the intrinsic growth rate of the host population is greater. In other words, vertical transmission tends to temper pathogen–host relationships when a pathogen’s capacity to spread via this mode increases. With this in mind, it makes sense that host-pathogen benevolence readily emerges in serial-passaging experiments (Bull et al., 1991; Bull and Molineux, 1992) that maintain exponential host population growth. It also makes sense that, in controlled lab settings, vertically transmitted viruses are more strongly favored when host fecundity increases (Pagán et al., 2014).

We also find that more costly immune responses often promote benign coevolutionary outcomes for pathogens and hosts. Certainly, a more expensive immune system limits the host’s ability to clear an infection. As a result, hosts unable to afford recovery could mitigate the detrimental effects of parasitism by tolerating infections, which could ultimately elicit an in-kind coevolutionary response from the pathogen. Examples of pathogens evolving to manipulate their hosts for personal gain abound (Ewald, 1994). Perhaps by looking for the kind of cost-sensitive hosts envisioned by our model we can flip this theme on its head and find examples of hosts that manipulate pathogens.

Finally, we find that virulence tends to increase as the profitability of horizontal transmission rises, even when vertical transmission of the pathogen is possible. The change in virulence, here, is the result of alteration of the trade-off faced by the pathogen. It is related to the increase in virulence observed following the deployment of imperfect vaccines, when these vaccines reduce pathogen-induced mortality without limiting horizontal transmission (Fleming-Davies et al., 2018; Read et al., 2015). Whereas imperfect vaccines reduce the costs of horizontal transmission faced by pathogens without changing the benefit (Gandon et al., 2001), our prediction is the consequence of increasing the benefit of horizontal transmission without changing the cost.

Our results are limited by the fact we have ignored density-dependent growth of the host population. This feature allows us to follow other authors and model coevolution as a game played only between a host and its pathogen (Day and Burns, 2003; Taylor et al., 2006; van Baalen, 1998), rather than a more complicated one played among mutants and residents of both species, as is commonly found in adaptive-dynamics models (Dieckmann & Law, 1996). The assumption also means that pathogen-induced mortality is the sole factor keeping host-population growth in check. Practically speaking, then, if pathogens were to become perfectly benign, our model would need to be revised. It follows that our predictions concerning the runaway reciprocated de-escalation must, in a strict sense, be viewed as a signal that such de-escalation is only a possibility. The simplifications we have made here, though helpful in many respects, also prevent us from estimating virulence measures in important (non-equilibrium) scenarios. This leaves open the question of what epidemiological patterns we expect to see when pathogen–host relationships de-escalate as much as possible. Future work can address this shortcoming with more complicated models and methods of analysis.

Another important pair of assumptions involve mutation. First, when building the fitness functions that serve as the foundation of our model, we allow hosts to be infected by no more than one strain of pathogen, and this limits our ability to generalize. In reality, a host could be infected by multiple pathogen strains because several strains happen to be circulating in the population or because a mutant strain has arisen de novo within the host. Within-host pathogen diversity can accelerate the evolution of increased virulence, for example when host immunity selects for more aggressive strains who can “win the race” to create new infections (Mackinnon & Read, 2004). Of course, within-host pathogen diversity can also lead to lower virulence when co-infecting strains are related (Frank, 1992), and so further modeling is required before we can assess how limited we are by this first assumption.

Second, we assume the mutations that arise in the host population are not correlated with those that arise in the pathogen population. Evolutionary theories of cooperation tell us that opposing interests can be brought into alignment by increasing the phenotypic correlations of the parties involved (Fletcher & Doebeli, 2009). Consequently, it is possible that a model in which such correlations are allowed to build would predict lower virulence with vertical transmission. Nevertheless, what we have demonstrated is that vertical transmission, alone, does not necessitate such a reduction in virulence.

Definitions of virulence found in the literature vary (Day, 2002; Dieckmann et al., 2002). Consequently, one may wonder, are the points we raise here novel only because our concept of virulence diverges substantially from those used in earlier studies of vertical transmission? Our response to this question is, no. Much previous work on vertical transmission recognizes that virulence is a reduction in overall host fitness, reflected in changes to the survival and fecundity of infected hosts (Bull et al., 1991; Bull and Molineux, 1992; Lipsitch et al., 1995, 1996; Stewart et al., 2005). The measure of virulence found in our Equation 4 also describes the reduction in host survival and fecundity owing to infection. While we go a step further than previous authors by using a virulence measure that correctly accounts for reduction in offspring quantity and quality, any rise in virulence we observe is due to evolutionary responses by the host. All else being equal, vertical transmission is certainly expected to select for pathogens with lower virulence; however, in a coevolutionary setting we cannot change vertical transmission rates and expect all else to remain equal. This complicated interplay is on display here.

## Supplementary material

Supplementary material is available online at *Evolution Letters*.

## Data availability

Code used to generate numerical results accompanies the submission and is included in Supplementary Material.

## Author contributions

G.W. and F.U. conceived the study. G.W. and G.S. developed and analyzed the model. G.S. wrote the computer code. G.S. and G.W. drafted the initial version of the manuscript and all authors contributed to later versions.

*Conflict of interest*: The authors declare no conflict of interest.

## Acknowledgments

We are grateful for the comments provided by two anonymous reviewers. This work was supported by the Natural Sciences and Engineering Research Council of Canada.

## Supplementary Material

qrad028_suppl_Supplementary_Material
