# Supplementary material for: Vertical transmission does not always lead to benign pathogen–host associations
Source: Evol Lett. 2023 Aug 10;7(5):305–14. doi: 10.1093/evlett/qrad028 (PMC10565904; doi:10.1093/evlett/qrad028)
Supplement: qrad028_suppl_Supplementary_Material [file qrad028_suppl_supplementary_material.zip › Supplement/Supplementary Text.pdf]

Supplementary Material for, “Vertical transmission does not always lead to benign pathogen-host associations”

George Shillcock[1], Francisco Úbeda[2], and Geoff Wild[1]

[1] Dept of Mathematics, Western University, London, Ontario, Canada

[2] Dept of Biological Sciences, Royal Holloway University of London, Egham, Surrey, UK

Contents

A Population Dynamics **1**

B Fitness **3**

B.1 Preamble 3

B.2 Pathogen fitness 4

B.3 Host fitness 5

C Methods of Analysis **7**

C.1 Co-evolution from evolutionary game theory 7

C.2 Alternative game-theoretic approach 8

C.3 Specific forms of transmissibility and host reproduction 12

C.3.1 Complete vertical transmission 13

C.4 Numerical methods 17

A. Population Dynamics

We start with a standard model for the dynamics of a host population in the presence of an infectious, obligate micropathogen. Let $S = S(t)$ denote the number of host individuals not infected by the pathogen at time t. Although they are not currently infected, these hosts are susceptible to

infection in the future. Let $I = I(t)$ denote the number of hosts infected by the pathogen at time t. Our immediate aim is to describe the change in S and I over time and arrive at a prediction about long-term dynamic behaviour of the system.

Hosts die and reproduce. We use $\mu > 0$ to denote the constant per-capita mortality rate of susceptible hosts. Infection increases the per-capita mortality rate of hosts infected by a pathogen by α. We use b_S and b_I to denote the per-capita birth rate of susceptible and infected hosts, respectively. To ensure the population size reaches some positive equilibrium we assume $b_S > \mu$ (birth rate of susceptible individuals exceeds their rate of death) and $b_I < \mu + \alpha$ (death rate of infected individuals exceeds their rate of birth). To be clear, host population growth in this model is kept in check by the pathogen. This is a common assumption in the literature [1, 2]. It simplifies the analysis while keeping attention focused on the tension between host and pathogen.

Infected hosts can transmit the pathogen horizontally to their susceptible counterparts, and vertically to their newborn offspring. We model the total rate at which horizontal transmission occurs as $\beta\, S\, I$, where $\beta > 0$ reflects the transmissibility of the pathogen. We capture vertical transmission by assuming an offspring produced by an infected individual is also infected when it is born with probability v (the offspring is born susceptible with probability $1 - v$). Importantly, we also assume that infected hosts can clear their pathogen, and we use γ to denote the per-capita rate at which this occurs. When an infection is cleared, however, the recovered individual is immediately susceptible to new infection.

We use the following system of differential equations to capture the population dynamics described above:

$$\begin{aligned} S' &= b_S\, S + (1 - v)\, b_I\, I - \beta\, S\, I - \mu\, S + \gamma\, I, \\ I' &= v\, b_I\, I + \beta\, S\, I - (\mu + \gamma + \alpha) I. \end{aligned} \tag{A.1}$$

If the number of susceptible individuals is equal to

$$\bar{S} = \frac{\mu + \gamma + \alpha - v\, b_I}{\beta} \tag{A.2}$$

and the number of infected individuals is equal to

$$\bar{I} = \frac{b_S - \mu}{\mu + \alpha - b_I}\, \frac{\mu + \gamma + \alpha - v\, b_I}{\beta}, \tag{A.3}$$

then the population is at equilibrium and parasitic infections are endemic. On a related point, our requirement that $b_S > \mu$ and $b_I < \mu + \alpha$ implies that $\bar{S}$ and $\bar{I}$ are both positive.

The local asymptotic stability of the equilibrium state, represented by $\bar{S}$ and $\bar{I}$, can be determined by considering the Jacobian matrix arising from (A.1). When evaluated at equilibrium, that matrix is given by

$$J = \begin{bmatrix} b_S - (\beta\bar{I} + \mu) & (1-v)\,b_I + \gamma - \beta\bar{S} \\ \beta\,\bar{I} & v\,b_i + \beta\bar{S} - (\mu + \gamma + \alpha) \end{bmatrix}$$

$$= \begin{bmatrix} -(b_S - \mu)\frac{(1-v)b_I+\gamma}{\mu - b_I} & -(\mu + \alpha - b_I) \\ \beta\bar{I} & 0 \end{bmatrix}.$$

The trace of J is negative, and the determinant of J is positive. Therefore, the Routh-Hurwitz criteria are satisified and we can conclude that the equilibrium $\bar{S}, \bar{I}$ is locally asymptotically stable.

B. Fitness

B.1 Preamble

To model the co-evolution of pathogen and host we must express the fitness of each, mathematically. In this section, we describe the fitness of a pathogen lineage and that of a host lineage, at the equilibrium represeted by $\bar{S}$ and $\bar{I}$. Our approach generalizes the one laid out by Day and Burns [1] by allowing for vertical transmission of the pathogen.

We define fitness in terms of the long-term success of a genetic lineage. If a given lineage represents a portion of an equilibrium population that is growing, then it will be favoured by selection. By contrast, if a given lineage represents a portion of an equilibrium population that is shrinking, then it will be disfavoured by selection. In all cases we assume that lineages are made up of haploid asexual individuals, but we expect the same predictions from a model with sexual individuals and additive interactions between alleles of the same locus [3].

The fitness of a given pathogen lineage depends on, among other things, the additional mortality its members impose on a host during infection. When considering pathogen fitness, we assume that the infection-induced mortality, previously denoted α, is genetically determined and necessary for vertical transmission. To capture this we treat the transmissibility β as an increasing function of α. We write β, now, as $\beta(\alpha)$ with $\beta'(\alpha) > 0$ and add the additional assumption that $\beta''(\alpha) < 0$. Implicit here is the requirement that hosts are infected by one (and only one) pathogen lineage. In other words, we are ignoring super-infection implicitly when determining pathogen fitness.

The fitness of a given host lineage depends on, among other things, the rate at which its members rid themselves of pathogens when infected. When considering host fitness we assume that recovery, previously denoted γ, is genetically determined and reduces the host's ability to reproduce while infected. To capture this we treat b_I, the birth rate of infected hosts, as a decreasing function of recovery. We write b_I, now, as $b_I(\gamma)$ with $b_I'(\gamma) < 0$ and $b_I''(0) \leq 0$. Implicit here is the assumption that the ability to recover from infections imposes no cost on a host while it is susceptible. In other words, we are focused on the evolution of only those parts of the host's immune system that are activated after infection has occurred.

We now focus our attention on developing explicit expressions for fitness. We tackle the pathogen and the host, in turn, in the subsections below.

B.2 Pathogen fitness

We suppose a rare lineage of mutant pathogens arises in a resident population of hosts and pathogens that had been at equilibrium ($\bar{S}$ and $\bar{I}$). We assume further that members of the mutant pathogen lineage induce infection-related host mortality at rate $\hat{\alpha}$, in contrast to their more common resident counterparts who still induce mortality at rate α. To be clear, we are allowing for mutant pathogens here, but the host population remains genetically homogeneous.

While it is rare, the dynamics of the mutant pathogen lineage can be captured by

$$\hat{I}' = \big(\beta(\hat{\alpha})\,\bar{S} - (\hat{\alpha} + \mu + \gamma - v\,b_I(\gamma))\big)\,\hat{I}, \tag{B.4}$$

where $\hat{I} = \hat{I}(t)$ denotes the number of hosts infected by the mutant pathogen at time t, and where $\bar{S}$ is still given by equation (A.2) (but now $\beta = \beta(\alpha)$ and $b_I = b_I(\gamma)$). The mutant pathogen lineage is growing when the factor multiplying $\hat{I}$ in (B.4) is positive. The mutant lineage is tending toward extinction when the same factor is negative. Therefore, we can use

$$W_p(\hat{\alpha}, \alpha) = \beta(\hat{\alpha})\,\bar{S} - (\hat{\alpha} + \mu + \gamma - v\,b_I(\gamma))$$

as pathogen fitness. We can say the mutant pathogen lineage is favoured by selection when $W_p > 0$ and is disfavoured when $W_p < 0$. The definition of $\bar{S}$ as an equilibrium tells us that the mutant lineage is neither favoured nor disfavoured ($W_p = 0$) when it is phenotypically indistinguishable from the resident pathogen (i.e. in the case where $\hat{\alpha} = \alpha$). It turns out that it is equivalent to assert

that the mutant pathogen invades when

$$\frac{\beta(\hat{\alpha})}{\mu + \hat{\alpha} + \gamma - v\, b_I(\gamma)} > \frac{\beta(\alpha)}{\mu + \alpha + \gamma - v\, b_I(\gamma)} \tag{B.5}$$

and is eliminated when the equality is reversed. From this re-statement it is clear that selection on the pathogen will act to maximize

$$w_p(\alpha, \gamma) = \frac{\beta(\alpha)}{\mu + \alpha + \gamma - v\, b_I(\gamma)} \tag{B.6}$$

in infection-induced mortality α.

Generically, in this model when a rare mutant is favoured by selection its advantage will persist and carry it on to fixation [4]. In other words, a rare mutant that successfully invades will, under a broad range of conditions, eliminate its competitor and become the new resident. It this process of repeated invasion and fixation of new mutants by which we envision evolution of the pathogen occurs.

B.3 Host fitness

We now suppose that a rare lineage of mutant hosts has arisen in a resident population that had been at equilibrium (again, $\bar{S}$ and $\bar{I}$). We assume mutant hosts recover from infections at rate $\tilde{\gamma}$ in contrast to their resident counterparts who recover at rate γ. To be clear, there is now genetic diversity among hosts but the pathogen population is genetically homogeneous.

With the above quantities in mind, the dynamics of the mutant-host lineage are captured by

$$\begin{bmatrix} \tilde{S}' \\ \tilde{I}' \end{bmatrix} = \begin{bmatrix} b_S - (\beta\, \bar{I} + \mu) & (1 - v)\, b_I(\tilde{\gamma}) + \tilde{\gamma} \\ \beta\, \bar{I} & v\, b_I(\tilde{\gamma}) - (\tilde{\gamma} + \mu + \alpha) \end{bmatrix} \begin{bmatrix} \tilde{S} \\ \tilde{I} \end{bmatrix} \tag{B.7}$$

where $\tilde{S} = \tilde{S}(t)$ and $\tilde{I} = \tilde{I}(t)$ give the number of susceptible and infected mutant hosts, respectively, at time t. Here, $\bar{I}$ is still given by equation (A.3), but now $\beta = \beta(\alpha)$ and $b_I = b_I(\gamma)$.

The mutant lineage of hosts is growing when at least one of the eigenvalues of the matrix in (B.7) has positive real part. The diagonal elements of the matrix are both negative; in particular, we require that $\tilde{\gamma}$ be ecologically feasible, meaning $b_I(\tilde{\gamma}) < \mu + \alpha$ (this inequality will hold, by continuity, for $\tilde{\gamma}$ sufficiently close to γ). Therefore, the Routh-Hurwitz criteria tell us that the fate of the mutant host is determined solely by the determinant of the matrix (B.7). When the

determinant of the matrix is positive, the mutant lineage is tending toward extinction, and when the determinant is negative the mutant lineage is growing. We can, therefore, use

$$W_h(\tilde{\gamma}, \gamma) = (b_S - \mu)\big(\tilde{\gamma} - v\, b_I(\tilde{\gamma}) + \mu + \alpha\big) - \beta(\alpha)\, \bar{I}\, \big(\mu + \alpha - b_I(\tilde{\gamma})\big)$$

(the determinant multiplied by -1) as host fitness. Selection favours the mutant host when $W_h > 0$, and selection disfavours the mutant host when $W_h < 0$. As expected, if the mutant cannot be distinguished phenotypically from the resident ($\tilde{\gamma} = \gamma$), then $W_h = 0$ and it is neither favoured nor disfavoured by selection. As with the pathogen, the action of selection on the host can be restated as a maximization problem. In this case, we see that a mutant host invades when

$$\frac{\mu + \alpha + \tilde{\gamma} - v\, b_I(\tilde{\gamma})}{\mu + \alpha - b_I(\tilde{\gamma})} > \frac{\mu + \alpha + \gamma - v\, b_I(\gamma)}{\mu + \alpha - b_I(\gamma)} \tag{B.8}$$

and it is eliminated when the inequality is reversed. Selection, therefore, acts to maximize

$$w_h(\gamma, \alpha) = \frac{\mu + \alpha + \gamma - v\, b_I(\gamma)}{\mu + \alpha - b_I(\gamma)} \tag{B.9}$$

in γ. Again, a successful rare mutant is expected to eliminate the existing resident to become the new established host strain in the population. Like the pathogen, then, the host evolves through a repeated process of mutation and fixation events.

In the main text, we measure pathogen virulence as a reduction in host fitness owing to infection. We can develop an expression for virulence consistent with this definition by subtracting the lifetime reproductive success of a host that is currently infected from that of a host that is currently susceptible. Equivalently, we can subtract the reproductive value of an infected host from that of a susceptible host, which is what we actually do. Reproductive values of individual host types come from a consideration of the matrix in (B.7) with $\tilde{\gamma} = \gamma$. In that case the quasi-positive nature of the matrix, and the definition of the equilibrium state guarantee that the matrix, itself, has a dominant zero eigenvalue. Reproductive values of individual host types, then, correspond to the entries of the left eigenvector associated with the dominant zero eigenvalue. Setting the reproductive value of a susceptible host equal to one, we find that of an infected host to be

$$\frac{(1 - v) b_I(\gamma) + \gamma}{\gamma + \mu + \alpha - v\, b_I(\gamma)}. \tag{B.10}$$

Subtracting this quantity from 1 gives us the expression in Eq (4) of the main text.

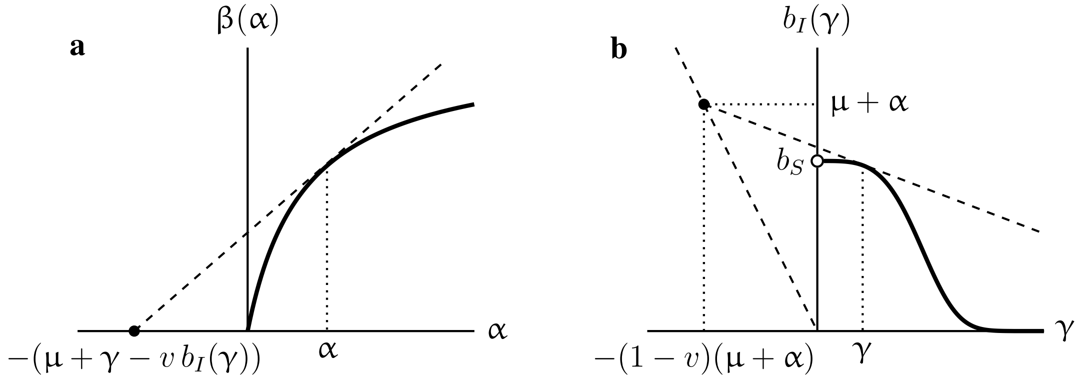

Figure C.1: The geometric construction that illustrates the solution to the co-evolutionary equilibrium condition (C.11) (also system 2 in the main text). As we explain in Appendix C.1, the solution is both convergence stable and evolutionarily stable whenever $\beta''(\alpha) < 0$ and $b_I''(\gamma) < 0$, as illustrated. Panel **a** shows that increasing γ will move α to the right, all else being equal. Panel **b** shows that increase α will move γ to the right, all else being equal. Similar geometric constructions can be found throughout the literature [5, 6].

C. Methods of Analysis

C.1 Co-evolution from evolutionary game theory

We assume mutation events are so rare that mutants of one species arise and are fixed before additional mutants of either species appear. That said, the co-evolutionary process will be at equilibrium when α and γ satisfy

$$\left.\frac{\partial W_p}{\partial \hat{\alpha}}\right|_{\hat{\alpha}=\alpha,\tilde{\gamma}=\gamma} = 0 \;\leftrightarrow\; \partial_\alpha w_p(\alpha,\gamma) = 0, \tag{C.11a}$$

$$\left.\frac{\partial W_h}{\partial \tilde{\gamma}}\right|_{\hat{\alpha}=\alpha,\tilde{\gamma}=\gamma} = 0 \;\leftrightarrow\; \partial_\gamma w_h(\gamma,\alpha) = 0. \tag{C.11b}$$

We will refer to equations (C.11) as the co-evolutionary equilibrium conditions for the pathogen (C.11a) and host (C.11b), respectively. The co-evolutionary equilibrium condition for the pathogen, in equation (C.11a), rearranges to equation (2a) in the main text. The co-evolutionary equilibrium condition for the host, in equation (C.11b), rearranges to equation (2b) in the main text. It is useful to observe that the co-evolutionary equilibrium conditions can be represented geometrically by the illustrations provided in Figure C.1.

In keeping with Day and Burns [1], we will say trait pairs that satisfy (C.11) are convergence stable whenever they satisfy

$$\frac{d}{d\alpha}\left[\left.\frac{\partial W_p}{\partial \hat{\alpha}}\right|_{\hat{\alpha}=\alpha,\tilde{\gamma}=\gamma}\right] < 0 \quad \text{and} \quad \frac{d}{d\gamma}\left[\left.\frac{\partial W_h}{\partial \tilde{\gamma}}\right|_{\hat{\alpha}=\alpha,\tilde{\gamma}=\gamma}\right] < 0. \tag{C.12}$$

Carrying out the differentiation indicated in the previous line, we see the convergence-stability conditions simplify to $\beta''(\alpha) < 0$ and $b_I''(\gamma) < 0$.

Again in keeping with Day and Burns [1], we say trait pairs that satisfy (C.11) are evolutionarily stable whenever

$$\left.\frac{\partial^2 W_p}{\partial \hat{\alpha}^2}\right|_{\hat{\alpha}=\alpha,\tilde{\gamma}=\gamma} < 0, \quad \text{and} \quad \left.\frac{\partial^2 W_h}{\partial \tilde{\gamma}^2}\right|_{\hat{\alpha}=\alpha,\tilde{\gamma}=\gamma} < 0. \tag{C.13}$$

It is straightforward to show that evolutionary stability is guaranteed when, once again, we have $\beta''(\alpha) < 0$ and $b_I''(\gamma) < 0$.

In summary, we can consider any solution (γ, α) to equations (C.11) for which $\beta''(\alpha) < 0$ and $b_I''(\gamma) < 0$ to be a stable according to the definitions provided so far. Given that the evolution of the pathogen and host, respectively, can be modelled as separate maximization problems, it is hardly surprising that the same second-derivative conditions guarantee both convergence stability and evolutionary stability. When written in terms of alternative fitness functions w_p and w_h, it is clear that both (C.12) and (C.13) amount to $\partial_\alpha^2 w_p < 0$ and $\partial_\gamma^2 w_h < 0$.

C.2 Alternative game-theoretic approach

We now modify the approach and model our co-evolutionary problem as a non-cooperative game played, in isolation, by a pathogen and the host that supports it. This approach corresponds to the presentation of the problem in the main text.

We view the trait expressed by a given player as its strategy. That said, the goal of the pathogen player is to maximize $w_p(\alpha, \gamma)$ in its strategy (α), while the host player's goal is to maximize $w_h(\alpha, \gamma)$ in its strategy (γ).

To solve the game we use best-response functions. The pathogen's best response to the host is defined as

$$\alpha_{\text{br}}(\gamma) = \underset{\alpha}{\text{argmax}}\left\{\frac{\beta(\alpha)}{\mu + \alpha + \gamma - v\, b_I(\gamma)}\right\}$$

but can also be found by solving equation (C.11a) for α. Similarly, the host's best response to the

pathogen is

$$\gamma_{\rm br}(\alpha) = \underset{\gamma}{\rm argmax} \left\{ \frac{\mu + \alpha + \gamma - v\, b_I(\gamma)}{\mu + \alpha - b_I(\gamma)} \right\}$$

but can also be found by solving to equation (C.11b) for γ.

We generate solutions by iterating a composed best-response map; for example $\gamma_{\rm br} \circ \alpha_{\rm br}$. Equilibrium outcomes are fixed points of the composed best-response maps; in other words, points at which best-response curves intersect. An equilibrium outcome, (γ^*, α^*) is stable whenever $|(\gamma_{\rm br} \circ \alpha_{\rm br})'(\gamma^*)| < 1$, i.e. whenever it attracts nearby trajectories generated by the iterative process. The geometric constructions in Figure C.1 show that best-response functions are increasing in their respective arguments. Consequently, the stability criterion simplifies to

$$\gamma'_{\rm br}(\alpha^*)\, \alpha'_{\rm br}(\gamma^*) < 1 \;\leftrightarrow\; \alpha'_{\rm br}(\gamma^*) < \frac{1}{\gamma'_{\rm br}(\alpha^*)} \tag{C.14}$$

where we have also applied the chain rule when simplifying. A graphical interpretation of the stability condition is presented in Figure 1 of the main text. It shows the graph of $\alpha_{\rm br}$ and $\gamma_{\rm br}$ plotted in γ, α-space. From that figure we see that stability requires the $\alpha_{\rm br}$ curve pass through the equilibrium point with a slope that is not as steep as that with which $\gamma_{\rm br}$ (or rather the reflection of this in the diagonal) passes through the same point. An equilibrium outcome is unstable whenever the inequality in (C.14) is reversed. In this case the equilibrium repels trajectories and separates basins of attraction of alternative solutions. In that sense, an unstable equilibrium acts as a threshold (Figure 1, main text)

In general, we also expect to find non-equilibrium ‘runaway’ solutions to the game. In those cases, trajectories traced out by iterating the composed best-response maps lead parties to escalate or de-escalate in a reciprocated manner (Figure 1, main text).

At this point, it makes sense to ask: how do the outcomes predicted by our alternative game-theory model compare to outcomes predicted by the evolutionary game-theory model presented in the previous section? The key difference is that the previous approach considers any intersection between best-response curves to be stable: all solutions to (C.11) are both convergence stable and evolutionarily stable. In contrast, under the simple approach intersections between best-response curves are not always considered stable. Indeed, the stability condition laid out in line (C.14) is different from both conditions (C.12) and (C.13).

We can develop the stability condition in (C.14) further using system (2) from the main text.

The pathogen's best response satisfies equation (2a), whereas the host's best response satisfies (1b). Implicit differentiation of (2a) gives us

$$\alpha'_{\mathrm{br}}(\gamma^*) = -\frac{\beta'(\alpha^*)}{\beta''(\alpha^*)}\,\frac{1 - v\,b'_I(\gamma^*)}{\mu + \alpha^* + \gamma^* - v\,b_I(\gamma^*)} > 0$$

and implicit differentiation of (2b) gives us

$$\gamma'_{\mathrm{br}}(\alpha^*) = \frac{b'_I(\gamma^*)}{b''_I(\gamma^*)}\,\frac{\gamma^* + (1-v)b_I(\gamma^*)}{((\mu+\alpha^*)(1-v)+\gamma^*)(\mu+\alpha^* - b_I(\gamma^*))} > 0.$$

It follows that condition C.14 can be restated as

$$-\frac{\beta'(\alpha^*)}{\beta''(\alpha^*)}\,\frac{1 - v\,b'_I(\gamma^*)}{\mu + \alpha^* + \gamma^* - v\,b_I(\gamma^*)} < \frac{b''_I(\gamma^*)}{b'_I(\gamma^*)}\,\frac{((\mu+\alpha^*)(1-v)+\gamma^*)(\mu+\alpha^* - b_I(\gamma^*))}{\gamma^* + (1-v)b_I(\gamma^*)}.$$

This inequality is not implied by the stability condition outlined in the previous section, namely the pair of conditions $\beta''(\alpha^*) < 0$ and $b''_I(\gamma^*) < 0$. It is for this reason, then, that the co-evolutionary stability concept suggested by (C.14), and used in the main text, is distinct from the one that appears in Day and Burns [1]. In particular, under the stability concept suggested by (C.14) not every solution to (C.11) such that $\beta''(\alpha) < 0$ and $b''_I(\gamma) < 0$ will be considered to be stable.

The difference between the definition of stability suggested by (C.14) and that used by Day and Burns [1] can be summed up using terminology introduced in work by Kisdi [7]. Stability, as the term is used in Day and Burns [1], is more accurately described as 'isoclinic stability' [7]. By contrast, condition (C.14) implies dynamic stability and absolute convergence stability, as the term is defined in Kisdi [7]. We explain in the paragraphs below.

Our evolutionary analysis is consistent with an approach that assumes traits follow certain trajectories over evolutionary time. Let $\alpha(\tau)$ represent the trajectory of the pathogen trait over evolutionary time τ, and let $\gamma(\tau)$ represent the trajectory of the host trait over evolutionary time τ. Assuming mutations arise independently in the two species, we can model trajectories as solutions to

$$\alpha'(\tau) = k_p\,\partial_\alpha w_p(\alpha,\gamma), \quad \gamma'(\tau) = k_h\,\partial_\gamma w_h(\gamma,\alpha) \tag{C.15}$$

where k_p and k_h are constants that reflect the mutation rate of pathogen and host, respectively. If trajectories satisfying (C.15) are in a steady state, we have

$$\partial_\alpha w_p(\alpha,\gamma) = 0, \quad \partial_\gamma w_h(\gamma,\alpha) = 0.$$

The former equation defines the pathogen's best response as a function of γ (namely, $\alpha_{\mathrm{br}}(\gamma)$), while the latter defines the host's best response as a function of α (namely, $\gamma_{\mathrm{br}}(\alpha)$). Points of intersection between best-response curves, therefore, represent steady-state solutions of Eq (C.15).

The local asymptotic stability of steady-state solutions to Eq (C.15), denoted (α^*, γ^*), depends on the the matrix

$$J = \begin{bmatrix} \partial^2_{\alpha} w_p(\alpha^*, \gamma^*) & \partial^2_{\alpha\gamma} w_p(\alpha^*, \gamma^*) \\ \partial^2_{\alpha\gamma} w_h(\gamma^*, \alpha^*) & \partial^2_{\gamma} w_h(\gamma^*, \alpha^*) \end{bmatrix}.$$

Specifically, local asymptotic stability is guaranteed when the real part of every eigenvalue of J is negative. In this case, local asymptotic stability is guaranteed whenever

$$\mathrm{tr} J < 0, \quad \text{and} \quad \det J > 0.$$

The isoclinic conditions, which hold for our model, imply that the trace of J is negative, and so local asymptotic stability hinges on the sign of $\det J$. We claim that $\det J > 0$ is equivalent to our condition (C.14), and we prove this in two steps.

First, we observe that, for our model, the mixed partial derivatives found in J are positive. Specifically, we have

$$\partial^2_{\alpha\gamma} w_p(\alpha^*, \gamma^*) = \frac{\beta(\alpha^*)}{((1-v)(\mu+\alpha^*)+\gamma^*)\,(\mu+\alpha^*+\gamma^*-v\,b_I(\gamma^*))^2} > 0$$

and

$$\partial^2_{\alpha\gamma} w_h(\gamma^*, \alpha^*) = \frac{(1-v)b_I(\gamma^*)+\gamma^*}{((1-v)(\mu+\alpha^*)+\gamma^*)\,(\mu+\alpha^*-b_I(\gamma^*))^2} > 0.$$

Second, we differentiate the equation $\partial_{\alpha} w_p(\alpha, \gamma) = 0$ implicitly with respect to γ and evaluate at the intersection point of the best response curves (i.e. the steady state of Eq C.15) to obtain

$$\alpha'_{\mathrm{br}}(\gamma^*) = -\frac{\partial^2_{\alpha\gamma} w_p(\alpha^*, \gamma^*)}{\partial^2_{\alpha} w_p(\alpha^*, \gamma^*)}.$$

After applying a similar procedure to $\partial_{\gamma} w_h(\gamma, \alpha) = 0$ we obtain

$$\gamma'_{\mathrm{br}}(\alpha^*) = -\frac{\partial^2_{\gamma\alpha} w_h(\gamma^*, \alpha^*)}{\partial^2_{\gamma} w_h(\gamma^*, \alpha^*)}.$$

Condition (C.14) can now be restated as

$$-\frac{\partial^2_{\alpha\gamma} w_p(\alpha^*, \gamma^*)}{\partial^2_{\alpha} w_p(\alpha^*, \gamma^*)} < -\frac{\partial^2_{\gamma} w_h(\gamma^*, \alpha^*)}{\partial^2_{\gamma\alpha} w_h(\gamma^*, \alpha^*)}.$$

Keeping in mind that $\partial^2_{\alpha} w_p(\alpha^*, \gamma^*)$ is negative and $\partial^2_{\gamma\alpha} w_h(\gamma^*, \alpha^*)$ is positive, we can re-write the previous condition as

$$-\partial^2_{\alpha\gamma} w_p(\alpha^*, \gamma^*)\, \partial^2_{\gamma\alpha} w_h(\gamma^*, \alpha^*) > -\partial^2_{\gamma} w_h(\gamma^*, \alpha^*)\, \partial^2_{\alpha} w_p(\alpha^*, \gamma^*) \tag{C.16}$$

which is equivalent to $\det J > 0$, as claimed. It follows that condition (C.14) is equivalent to a dynamic definition of convergence stability (see, for example, [8]). Because the mixed partial derivatives in (C.16) are positive, this condition is equivalent to the one for absolute convergence stability found in Eq 11 of [7]. Thus, the solutions to host-pathogen game that we present are dynamically stable, because they attract "extreme" trajectories followed by populations in which one party mutates more quickly than the other [7].

C.3 Specific forms of transmissibility and host reproduction

To make mathematical headway with the alternative game-theory approach we only study simple models for pathogen transmissibility. Specifically, we use $\beta(\alpha) = \alpha^n$, with $0 < n < 1$ (and so $\beta''(\alpha) < 0$). An interpretation of n comes from the observation that $n = d\log\beta(\alpha)/d\log\alpha$. This identity tells us that $n \times 100\%$ can be understood as the per-cent gain in horizontal transmission owing to a one per-cent increase in infection-induced mortality. Loosely speaking, n represents the horizontal transmission gains awarded to a pathogen who increases the mortality it inflicts on its host. A larger (resp. smaller) value of n suggests that horizontal transmission is a more (resp. less) attractive to a pathogen.

Tractability also requires a simple model of host reproduction. For host reproduction, then, we set $b_I(\gamma) = b_S - \lambda\gamma^2$, where $\lambda > 0$ and $b_I''(\gamma) < 0$ (when $\gamma > 0$). Here, λ represents the fecundity cost associated with increasing recovery rate. Greater λ means that recovery from infection is more costly and suggests the host should be more willing to tolerate infection. We note that $b_I(\gamma)$ could, in principle, be negative. As described in the main text, however, we can view this form of $b_I(\gamma)$ as an approximation of $b_S \exp\{-\lambda\gamma^2/b_S\}$ when λ is much smaller than b_S. With that constraint in mind, then, the quadratic form we have chosen is reasonable.

The simple functional forms of $\beta(\alpha)$ and $b_I(\gamma)$ we have chosen allow us to say specifically that

$$\alpha_{\text{br}}(\gamma) = \frac{n}{1-n}\left(\mu + \gamma - v\,\left(b_S - \lambda\gamma^2\right)\right), \tag{C.17a}$$

$$\gamma_{\text{br}}(\alpha) = -(1-v)(\mu+\alpha) + \sqrt{(1-v)^2(\mu+\alpha)^2 + \frac{\mu+\alpha-b_S}{\lambda}}. \tag{C.17b}$$

The forms of $\beta(\alpha)$ and $b_I(\gamma)$ we have chosen match those found in Day and Burns [1]. By setting $v = 0$ in equation (C.17) we obtain expressions that appear in Day and Burns [1], as expected (though C.25b does not appear in that paper). Furthermore, those same expressions are used to establish the solution to the game suggested by equation (2) of the main text for the case of no vertical transmission ($v = 0$).

C.3.1 Complete vertical transmission— We can also study, analytically, the case where vertical transmission is guaranteed ($v = 1$). When $v = 1$, best-response functions potentially intersect when γ is either

$$\gamma_1 = \frac{-n + \sqrt{8\,(n-1/2)\,(b_S-\mu)\,\lambda + n^2}}{4\,(n-1/2)\,\lambda}$$

or

$$\gamma_2 = \frac{-n - \sqrt{8\,(n-1/2)\,(b_S-\mu)\,\lambda + n^2}}{4\,(n-1/2)\,\lambda}.$$

The same expressions appear in equation (6) of the main text. Of course, $\alpha_{\text{br}}(\gamma_1)$ or $\alpha_{\text{br}}(\gamma_2)$ would give the pathogen coordinate of the corresponding point of intersection.

It is clear that γ_1 and γ_2 are complex whenever

$$8\left(n-\frac{1}{2}\right)(b_S-\mu)\lambda + n^2 < 0. \tag{C.18}$$

When (C.18) holds, then, best-response curves do not intersect and there can be no equilibrium solution to the game.

If $n < 1/2$ and (C.18) holds, then there is no equilibrium solution (as we have said). Furthermore, $\gamma_{\text{br}}(\alpha)$ lies above $\alpha_{\text{br}}(\gamma)$ when these are plotted in γ, α-space. It follows that that runaway reciprocated de-escalation is the predicted outcome (see Figure 1, main text). If $n < 1/2$ and (C.18) is reversed, then there are two intersections between the best response curves. In this case $\gamma_1 < \gamma_2$, and only γ_2 corresponds to a stable equilibrium as defined by (C.14). The pair $(\gamma_1, \alpha_{br}(\gamma_1))$ acts as a threshold that separates the stable equilibrium $(\gamma_2, \alpha_{\text{br}}(\gamma_2))$ from non-equilibrium runaway reciprocated de-escalation (Figure 1, main text). We want to stress here that had we adopted a notion of stability based on (C.12) or (C.13) we would have concluded that both $(\gamma_1, \alpha_{\text{br}}(\alpha_1))$ and $(\gamma_2, \alpha_{\text{br}}(\gamma_2))$ would have been categorized as stable.

As $n \to 1/2$ only γ_1 tends to a finite limit; that limit is $2(b_S - \mu)$. When $n = 1/2$, the pair $(\gamma_2, \alpha_{\text{br}}(\gamma_2))$ is lost. At the same time, the pair $(\gamma_1, \alpha_{\text{br}}(\gamma_1))$ still acts as a threshold, but now that threshold separates non-equilibrium runaway reciprocated escalation from non-equilibrium runaway reciprocated de-escalation (Figure 1, main text). We want to stress again that stability concepts not adopted here (and outlined in lines C.12 and C.13, above) would lead us to classify $(\gamma_1, \alpha_{\text{br}}(\gamma_1))$ as stable.

When $n > 1/2$ both γ_1 and γ_2 are real, but only γ_1 is positive. The positive pair $(\gamma_1, \alpha_{\text{br}}(\gamma_1)$ again acts as a threshold, that separates runaway reciprocated escalation from runaway reciprocated de-escalation (Figure 1, main text).

To supplement the investigation of the case $v = 1$ given in the main text we focus, for the moment, on subcases related to region B of Figure 1 of the main text. In region B, we find the threshold $(\gamma_1, \alpha_1) = (\gamma_1^*, \alpha_1^*)$ and the relatively escalated stable outcome represented by $(\gamma_2, \alpha_2) = (\gamma_2^*, \alpha_2^*)$. We connect more benign outcomes to changes in parameter combinations that increase the threshold separating mutual de-escalation from the stable outcome; i.e. changes that increase the (log of the) product $\alpha_1^* \gamma_1^*$. We also connect more benign outcomes to changes in parameter combinations that de-escalate the the stable outcome; i.e. that decrease the (log of the) product $\alpha_2^* \gamma_2^*$. As Figure C.2 shows, then, in region B more benign outcomes are once again promoted by lower n, larger $b_s - \mu$ and larger λ.

In region C of Figure 1 in the main text, only the threshold $(\gamma_1, \alpha_1) = (\gamma_1^*, \alpha_1^*)$ that separates mutual de-escalation from runaway mutual escalation exists. In this area, then, we associate more benign outcomes with increases in the threshold, as described above. Figure C.3 shows that benign outcomes here are promoted when n is lowered and $b_S - \mu$ raised. Notably, the cost parameter λ interacts with n and $b_S - \mu$, respectively, to affect co-evolutionary outcomes in region C. Whereas increases in cost elsewhere uniformly promote, the opposite is true in region C when n is large and/or $b_S - \mu$ is small (Figure C.3).

We can compare stable equilibrium outcomes for the $v = 0$ and $v = 1$ cases to get a sense of the effect of vertical transmission has at extreme ends of its range. Numerical computation of (α_0^*, γ_0^*) and (α_2^*, γ_2^*) using the expressions developed above (also Eqs 5 and 6 of the main text) show that stable equilibrium traits are expressed more strongly with complete vertical transmission ($v = 1$) (Table C.1). In addition, virulence measured as case mortality (Eq 3, main text) is slightly lowered

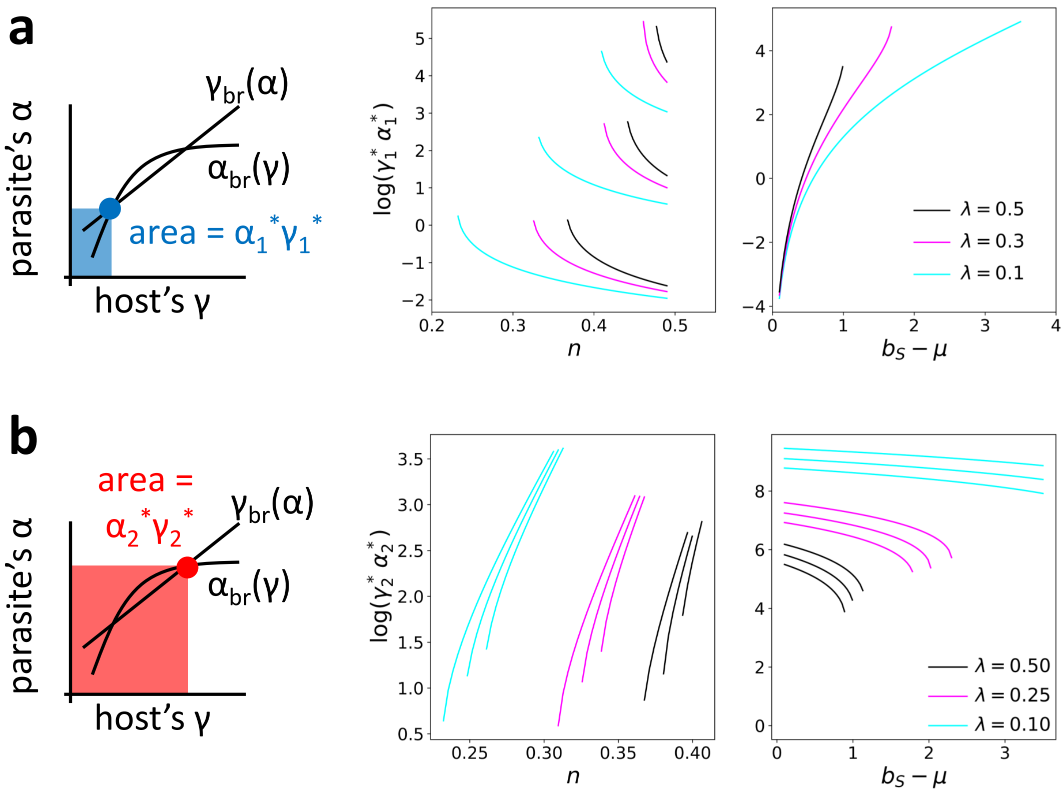

Figure C.2: Effect of model parameters on **a** threshold $(\gamma_1, \alpha_1) = (\gamma_1^*, \alpha_1^*)$ and **b** stable $(\gamma_2, \alpha_2) = (\gamma_2^*, \alpha_2^*)$ outcomes in region B identified in Figure 1 of the main text. Model parameters presented include return on horizontal transmission from induced host mortality (n), intrinsic growth rate of host population ($b_S - \mu$), and cost of host immune function (λ). Panel **a** center assumes $b_S - \mu = 0.25, 0.8, 2.25$, and right assumes $n = 0.45$ only. Panel **b** center assumes $b_S - \mu = 0.25, 0.30, 0.35$, and right assumes $n = 0.445, 0.450, 0.455$. In panel **a**, benign outcomes are promoted as $\alpha_1^* \gamma_1^*$ increases. In panel **b**, less aggressive outcomes are promoted as $\alpha_2^* \gamma_1^*$ decreases.

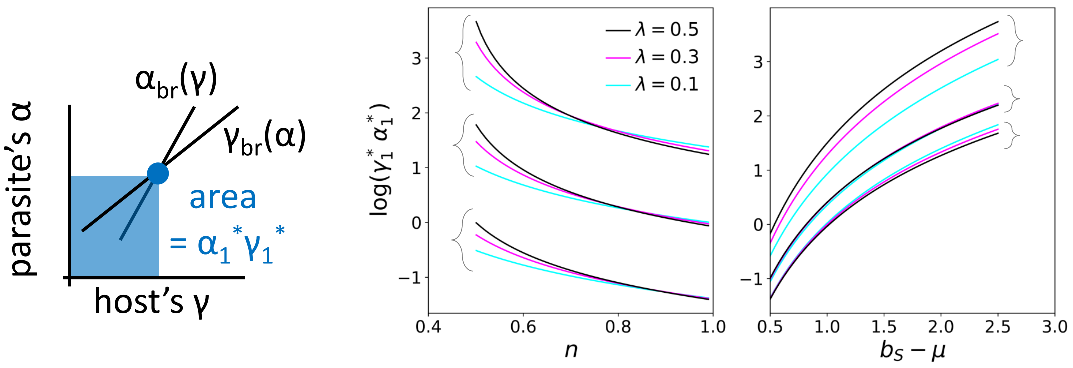

Figure C.3: Effect of model parameters on threshold $(\gamma_1, \alpha_1) = (\gamma_1^*, \alpha_1^*)$ in region C identified in Figure 1 of the main text. Model parameters presented include return on horizontal transmission from induced host mortality (n), intrinsic growth rate of host population ($b_S - \mu$), and cost of host immune function (λ). Center panel assumes $b_S - \mu = 0.5, 1, 2$, right panel assumes $n = 0.525, 0.75, 0.975$, and grey brackets are simply to guide visual interpretation of results. Benign outcomes are promoted as $\alpha_1 \gamma_1$ increases.

with vertical transmission, whereas virulence, measured as the fitness reduction experienced by the host (Eq 4, main text), is greater with complete vertical transmission (Table C.2).

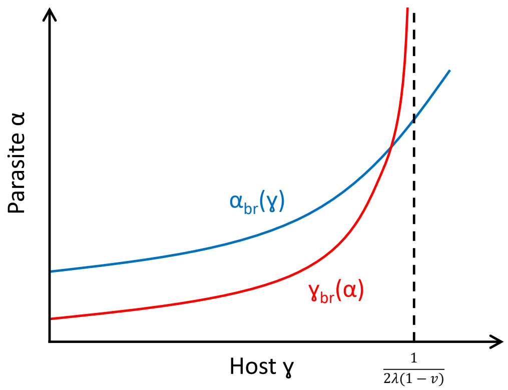

Figure C.4: Sketch of best-response curves in (C.17) laid out in γ, α-space. The graph of the host's best response $\gamma_{\rm br}(\alpha)$ (red) is always above the pathogen's best response $\alpha_{\rm br}(\gamma)$ (blue) in a neighbourhood of the vertical asymptote (dashed line). Though the sketch shows the curves intersecting once, this may not always be the case as explained in this supplement.

C.4 Numerical methods

In this subsection we outline how we find points of intersection between best response curves in (C.17) when $v < 1$. One can understand the outline we provide more readily if one first understands the geometry of the best-response curves themselves. The pathogen's best response curve in (C.17)a, for example, can be drawn as a concave-up parabola in γ, α-space (Figure C.4). The host's best response in (C.17)b, by contrast, is an increasing concave-down function in α, γ-space that saturates at $1/(2\lambda(1-v))$ with domain defined to ensure that $\gamma_{\rm br}(\alpha)$ is real (Figure C.4). It is important to notice also that the level at which $\gamma_{\rm br}$ saturates grows as $v \to 1$ from below; i.e. there is no such saturation in the special case $v = 1$ explored immediately above.

The picture of the best-response curves allows us to draw two qualitative conclusions about potential co-evolutionary outcomes when $v < 1$. First, the asymptote in Figure C.4 tells us that if best-response curves do not intersect, then $\gamma_{\rm br}(\alpha)$ must always lie above $\alpha_{\rm br}(\gamma)$. In this case, selection will lead both pathogen and host to de-escalate. Second, if intersections between the best-response curves occur, then the one nearest the asymptote will be stable, as described in line (C.14). Subsequent intersection points will alternate stability (unstable, stable, unstable, etc.) as

we move right-to-left from the stable intersection nearest the asymptote.

In the numerical method we use to find intersection points themselves, we first solve $\gamma = \gamma_{\text{br}}(\alpha)$ for α to obtain

$$\gamma_{\text{br}}^{-1}(\gamma) = \frac{\lambda\gamma^2 + 2\lambda\gamma(1-v)\mu + b_S - \mu}{1 - 2\lambda\gamma(1-v)} \tag{C.19}$$

If we graph this in γ, α-space we, of course, trace out the red curve seen in Figure C.4. It follows that the γ coordinate for a point of intersection between best-response functions solves $\alpha_{\text{br}}(\gamma) = \gamma_{\text{br}}^{-1}(\gamma)$, i.e. is a root of

$$\frac{n}{1-n}\left(\mu + \gamma - v\left(b_S - \lambda\gamma^2\right)\right)\left(1 - 2\lambda\gamma(1-v)\right) - \left(\lambda\gamma^2 + 2\lambda\gamma(1-v)\mu + b_S - \mu\right). \tag{C.20}$$

The roots of the expression in the previous line are also the roots of the cubic polynomial $p_0\gamma^3 + p_1\gamma^2 + p_2\gamma + p_3$ where

$$\begin{aligned} p_0 &= -\frac{2n\,\lambda^2\,(1-v)\,v}{1-n} \\ p_1 &= \frac{nv\lambda - 2n\lambda\,(1-v)}{1-n} - \lambda \\ p_2 &= \frac{n - 2n\lambda\,(1-v)\,(-b_S v + \mu)}{1-n} - 2\lambda\,(1-v)\,\mu \\ p_3 &= \frac{n\,(-b_S v + \mu)}{1-n} - b_S + \mu \end{aligned} \tag{C.21}$$

We used the coefficients to numerically estimate the the three roots of the cubic polynomial itself using Python v3.x—specifically, we applied the `roots` routine included with `numpy` to the array $[p_0, \ldots, p_3]$ (see iPython notebook `Shillcock et al.ipynb`). We recorded only the positive real roots we obtained and we verified $b_I(\gamma) > 0$ at each corresponding intersection point. As shown in Figure C.5, we would not have found a real positive intersection point for which the population-dynamical constraint $b_I(\gamma) < \mu + \alpha$ failed. As explained above, the largest of the real positive roots we found must correspond to a stable equilibrium outcome, the second largest would be an unstable threshold, and the smallest would be a stable equilibrium. We note that the numerical routine sometimes returns negative real roots, which is an artefactual result: a consequence of the fact that the domain of $\gamma_{\text{br}}^{-1}(\gamma)$ extends beyond the range of $\gamma_{\text{br}}(\alpha)$. We also note that we sometimes report unstable fixed points and no stable ones when $v < 1$. In those situations, the larger value of γ mean that the birth rate $b_I(\gamma)$ was negative and prompted us to disregard the result.

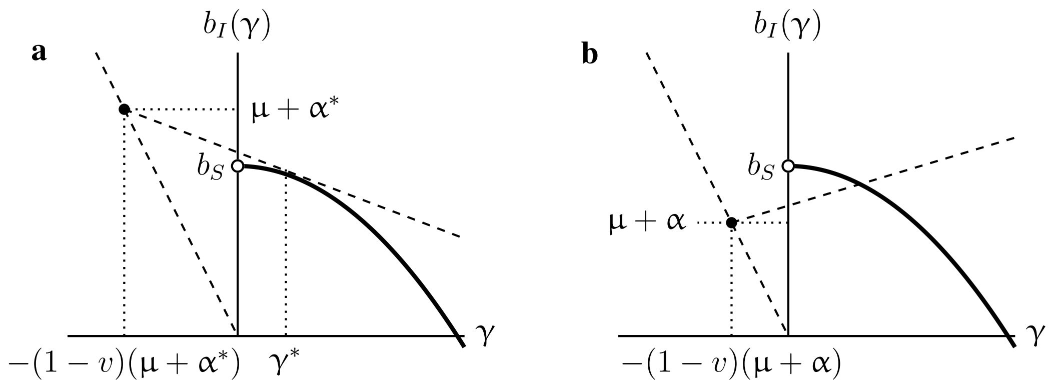

Figure C.5: When $b_I(\gamma) = b_S - \lambda\gamma^2$ we have confidence that $b_I(\gamma) < \mu + \alpha$ at equilibrium, as required by the population-dynamic model. Panel **a** shows that if (γ^*, α^*) represents a point of intersection between best-response curves with $\gamma^* > 0$, then $b_I(\gamma^*) \leq b_S < \mu + \alpha^*$. Panel **b** shows that we cannot find an intersection between best-response curves (γ, α) such that $\mu + \alpha < b_S$. When $b_I(\gamma) = b_S - \lambda\gamma^2$, we should, in principle, be concerned with $b_I(\gamma) < 0$. However, this will not be a valid concern for $\lambda \ll b_S$.

References

1. Day T, Burns J. 2003 A consideration of patterns of virulence arising from host-parasite coevolution. *Evolution* **57**, 671–676. (10.1111/j.0014-3820.2003.tb01558.x)

2. van Baalen M. 1998 Coevolution of recovery ability and virulence. *Proceedings of the Royal Society, B* **265**, 317–325. (10.1098/rspb.1998.0298)

3. Geritz SAH, Kisdi É. 2000 Adaptive dynamics in diploid, sexual populations and the evolution of reproductive isolation. *Proceedings of the Royal Society, B* **267**, 1671–1678. (10.1098/rspb.2000.1194)

4. Dercole F, Rinaldi S. 2008 *Analysis of Evolutionary Processes: The Adaptive Dynamics Approach and Its Applications*. Princeton, NJ: Princeton University Press.

5. Stearns SC, Medzhitov R. 2015 *Evolutionary Medicine*. Oxford, UK: Oxford University Press.

6. Úbeda F, Jansen VAA. 2016 The evolution of sex-specific virulence in infectious diseases. *Nature Communications* **7**, 13849. (10.1038/ncomms13849)

7. Kisdi É. 2006 Trade-off geometries and the adaptive dynamics of two co-evolving species.

Evolutionary Ecology Research **8**, 959–973.

8. Abrams PA, Matsuda H, Harada Y. 1993 Evolutionary unstable fitness maxima and stable fitness minima of continuous traits. *Evolutionary Ecology* **7**, 465–487. (doi.org/10.1007/BF01237642)

Table C.1: Comparison of stable equilibrium traits without vertical transmission (α_0^*, γ_0^*) and with vertical transmission (α_2^*, γ_2^*). Computations are carried out in the accompanying Python 3.x file, `Compare0and1.py`.

λ	n	$b_S - \mu$	α_0^*	α_2^*	γ_0^*	γ_2^*
0.050	0.150	0.100	0.456	0.699	1.583	3.460
0.050	0.150	0.114	0.440	0.659	1.491	3.301
0.050	0.150	0.128	0.422	0.613	1.390	3.116
0.050	0.150	0.141	0.402	0.558	1.276	2.886
0.050	0.150	0.155	0.378	0.478	1.145	2.541
0.050	0.200	0.252	0.663	1.493	1.653	4.983
0.050	0.200	0.266	0.633	1.435	1.532	4.837
0.050	0.200	0.279	0.596	1.372	1.384	4.675
0.050	0.200	0.293	0.545	1.302	1.179	4.491
0.010	0.100	0.155	0.860	1.297	6.738	10.685
0.010	0.100	0.169	0.839	1.268	6.550	10.486
0.010	0.100	0.183	0.817	1.239	6.349	10.277
0.010	0.100	0.197	0.793	1.208	6.133	10.057
0.010	0.100	0.210	0.766	1.175	5.896	9.823
0.010	0.100	0.224	0.737	1.141	5.630	9.573
0.010	0.100	0.238	0.703	1.103	5.324	9.303
0.010	0.100	0.252	0.661	1.063	4.947	9.006
0.010	0.100	0.266	0.600	1.018	4.404	8.673
0.015	0.100	0.100	0.598	0.871	4.381	7.171
0.015	0.100	0.114	0.579	0.843	4.208	6.974
0.015	0.100	0.128	0.558	0.813	4.019	6.761
0.015	0.100	0.141	0.534	0.781	3.809	6.529
0.015	0.100	0.155	0.508	0.745	3.569	6.271
0.015	0.100	0.169	0.476	0.705	3.280	5.978
0.015	0.100	0.183	0.432	0.658	2.890	5.627

Table C.1 continued.

λ	n	$b_S - \mu$	α_0^*	α_2^*	γ_0^*	γ_2^*
0.020	0.100	0.100	0.432	0.600	2.885	5.000
0.020	0.100	0.114	0.410	0.566	2.686	4.754
0.020	0.100	0.128	0.384	0.526	2.452	4.463
0.020	0.100	0.141	0.350	0.476	2.152	4.089
0.020	0.100	0.155	0.289	0.384	1.602	3.385
0.020	0.150	0.307	0.766	1.574	3.339	7.961
0.020	0.150	0.321	0.708	1.526	3.015	7.764
0.025	0.100	0.100	0.333	0.427	2.000	3.618
0.025	0.100	0.114	0.308	0.378	1.769	3.249
0.025	0.150	0.210	0.734	1.368	3.161	6.805
0.025	0.150	0.224	0.708	1.328	3.010	6.644
0.025	0.150	0.238	0.677	1.284	2.838	6.470
0.025	0.150	0.252	0.641	1.238	2.633	6.281
0.025	0.150	0.266	0.594	1.188	2.365	6.073
0.030	0.100	0.100	0.269	0.287	1.425	2.500
0.030	0.150	0.141	0.689	1.231	2.906	6.026
0.030	0.150	0.155	0.670	1.195	2.796	5.888
0.030	0.150	0.169	0.649	1.158	2.678	5.741
0.030	0.150	0.183	0.626	1.118	2.548	5.584
0.030	0.150	0.197	0.600	1.076	2.403	5.414
0.030	0.150	0.210	0.571	1.030	2.235	5.226
0.030	0.150	0.224	0.535	0.978	2.030	5.014
0.030	0.150	0.238	0.483	0.919	1.737	4.765

Table C.2: Comparison of virulence measures at stable equilibria with and without vertical transmission. Case mortality comes from Eq (3) of the main text, and Fitness Reduction (↓) comes from Eq (4) of the main text. As expected, case mortality with no vertical transmission = n [1]. Computations are carried out in the accompanying Python 3.x file, `Compare0and1.py`.

λ	n	$b_S - \mu$	case mort, $v = 0$	case mort, $v = 1$	Fitness ↓, $v = 0$	Fitness ↓, $v = 1$
0.050	0.150	0.100	0.150	0.135	0.158	0.257
0.050	0.150	0.114	0.150	0.133	0.149	0.248
0.050	0.150	0.128	0.150	0.130	0.139	0.238
0.050	0.150	0.141	0.150	0.126	0.128	0.224
0.050	0.150	0.155	0.150	0.119	0.114	0.203
0.050	0.200	0.252	0.200	0.200	0.165	0.333
0.050	0.200	0.266	0.200	0.197	0.153	0.326
0.050	0.200	0.279	0.200	0.195	0.138	0.319
0.050	0.200	0.293	0.200	0.192	0.118	0.310
0.010	0.100	0.155	0.100	0.100	0.135	0.176
0.010	0.100	0.169	0.100	0.099	0.131	0.173
0.010	0.100	0.183	0.100	0.099	0.127	0.170
0.010	0.100	0.197	0.100	0.098	0.123	0.167
0.010	0.100	0.210	0.100	0.098	0.118	0.164
0.010	0.100	0.224	0.100	0.097	0.113	0.161
0.010	0.100	0.238	0.100	0.097	0.106	0.157
0.010	0.100	0.252	0.100	0.096	0.099	0.153
0.010	0.100	0.266	0.100	0.095	0.088	0.148
0.015	0.100	0.100	0.100	0.096	0.131	0.177
0.015	0.100	0.114	0.100	0.096	0.126	0.173
0.015	0.100	0.128	0.100	0.095	0.121	0.169
0.015	0.100	0.141	0.100	0.094	0.114	0.164
0.015	0.100	0.155	0.100	0.093	0.107	0.158
0.015	0.100	0.169	0.100	0.092	0.098	0.152
0.015	0.100	0.183	0.100	0.090	0.087	0.144

Table C.2 continued.

λ	n	$b_S - \mu$	case mort, $v = 0$	case mort, $v = 1$	Fitness ↓, $v = 0$	Fitness ↓, $v = 1$
0.020	0.100	0.100	0.100	0.091	0.115	0.167
0.020	0.100	0.114	0.100	0.090	0.107	0.160
0.020	0.100	0.128	0.100	0.088	0.098	0.151
0.020	0.100	0.141	0.100	0.085	0.086	0.141
0.020	0.100	0.155	0.100	0.081	0.064	0.119
0.020	0.150	0.307	0.150	0.149	0.134	0.242
0.020	0.150	0.321	0.150	0.148	0.121	0.237
0.025	0.100	0.100	0.100	0.085	0.100	0.153
0.025	0.100	0.114	0.100	0.082	0.088	0.140
0.025	0.150	0.210	0.150	0.149	0.158	0.254
0.025	0.150	0.224	0.150	0.148	0.151	0.249
0.025	0.150	0.238	0.150	0.147	0.142	0.244
0.025	0.150	0.252	0.150	0.145	0.132	0.239
0.025	0.150	0.266	0.150	0.144	0.118	0.233
0.030	0.100	0.100	0.100	0.076	0.085	0.130
0.030	0.150	0.141	0.150	0.149	0.174	0.266
0.030	0.150	0.155	0.150	0.148	0.168	0.261
0.030	0.150	0.169	0.150	0.147	0.161	0.256
0.030	0.150	0.183	0.150	0.145	0.153	0.251
0.030	0.150	0.197	0.150	0.144	0.144	0.245
0.030	0.150	0.210	0.150	0.142	0.134	0.239
0.030	0.150	0.224	0.150	0.140	0.122	0.231
0.030	0.150	0.238	0.150	0.138	0.104	0.222
